# Identification and characterization of telocytes in rat testis

**DOI:** 10.18632/aging.102158

**Published:** 2019-08-14

**Authors:** Yifei Liu, Yu Liang, Siyi Wang, Imran Tarique, Waseem Ali Vistro, Haiyan Zhang, Abdul Haseeb, Noor Samad Gandahi, Adeela Iqbal, Tianci An, Huan Yang, Qiusheng Chen, Ping Yang

**Affiliations:** 1 MOE Joint International Research Laboratory of Animal Health and Food Safety, College of Veterinary Medicine, Nanjing Agricultural University, Nanjing 210095, Jiangsu, China; 2 School of Biological Engineering, Wuhu Institute of Technology, Wuhu 241003, Anhui, China; 3 College of Animal Science and Technology, Nanjing Agricultural University, Nanjing 210095, Jiangsu, China

**Keywords:** telocytes, telopodes, ultrastructure, CD34, PDGFRα

## Abstract

In this study, we investigated the localization, morphological features and cellular interactions of telocytes in the rat testicular interstitium. Transmission electron microscopy (TEM) and immunohistochemical and immunofluorescence analyses of the rat testicular interstitium showed a distinct layer of telocytes surround the seminiferous tubules along with inner layer of peritubular myoid cells. The majority of the telocytes were made up of a small cell body and moniliform prolongations that contained mitochondria and secretory vesicles. Some other telocytes were observed possessing large cell bodies. Within the testicular interstitium, the telocytes formed a network connecting peritubular myoid cells, Leydig cells as well as blood vessels. Immunohistochemical and double immunofluorescence analyses showed that rat testicular telocytes express CD34 and PDGFRα, but are negative for vimentin and α-SMA. Our findings demonstrate the presence of telocytes in the rat testicular interstitium. These cells interact with peritubular myoid cells, seminiferous tubules, Leydig cells and blood vessels via long telopode extensions, which suggests their vital role in the intercellular communication between different cell types within the rat testis.

## INTRODUCTION

Telocytes (TCs) are a type of interstitial cells that are found in several tissues [1]. They have elongated and moniliform processes called telopodes (Tps) with alternating thin (podomers) and dilated (podoms) segments [2], which distinguishes them from other cells [3]. Telopodes can also be dichotomous and form 3D networks with other tissue-resident cells anchored by hetero- and homo-cellular junctions [4]. TCs establish physical contact with other TCs, blood vessels [5], muscular tissue [6], stem cells [7], tissue resident cells [4], and nerve cells [8]. Although the actual functions of TCs is not clear, they may act as hormonal sensors in the female reproductive organs and may transmit slow waves generated by pacemaker ICC [9, 10]. In the male reproductive organs, the existence of TCs have been reported in the Chinese soft-shelled turtle and adult human testis. Testis is the male genital organ that produces sperms and testosterone, the male sex hormone [11, 12]. In rats and mice, the peritubular tissue is made up of a single layer of peritubular myoid cells (PMC). In rat testis, Losinno *et al* (2012) found that PMCs were shown to be surrounded by filaments consisting fibroblasts or vascular endothelial cells [13]. However, these filaments have not been studied in detail. Therefore, the cellular components of the peritubular tissue need further investigation. It has been demonstrated that PMCs, Sertoli cells (SC) and Leydig cells regulate spermatogenesis and testosterone production in an autocrine and paracrine manner [14–17], but the intercellular interactions between the PMC, SC and Leydig cells are not known [16, 18]. In a previous study, we identified and characterized TCs in the testis of the Chinese soft shelled turtle, *Pelodiscus sinensis* [19]. The aim of the present study was to identify TCs in the rat testis, and characterize their morphological features and interactions with other cells using transmission electron microscopy, immunohistochemistry, and immunofluorescence techniques.

## RESULTS

### Ultrastructural analysis of the telocytes in rat testicular interstitium

**Figure 1:**
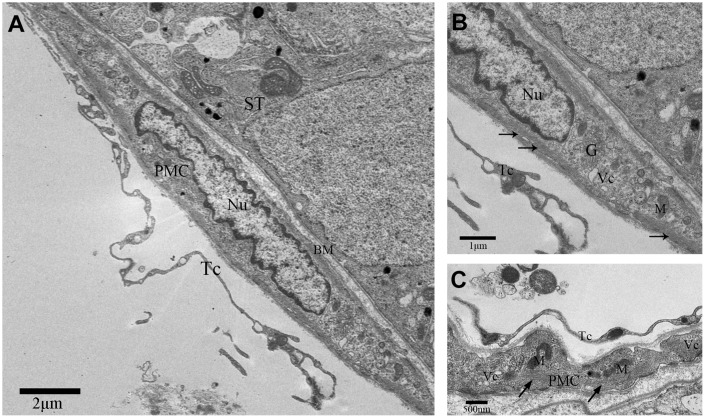
**TEM photograph of a peritubular myoid cell in the rat testis.** (**A**) Peritubular myoid cells are seen in the lamina propria of the seminiferous tubule. (**B**–**C**) A single peritubular myoid cell (PMC) is shown with actin filaments (indicated by the arrow), mitochondria, Golgi apparatus and secretory vesicles. Note: PMC: peritubular myoid cell; ST: seminiferous tubule; TC: telocyte; BM: basement membrane; Nu: nucleus; M: Mitochondria; G: Golgi apparatus;Vc: Vesicles. Scale Bar = **A**: 2μm; **B**: 1μm and **C**: 500nm.

**Figure 2:**
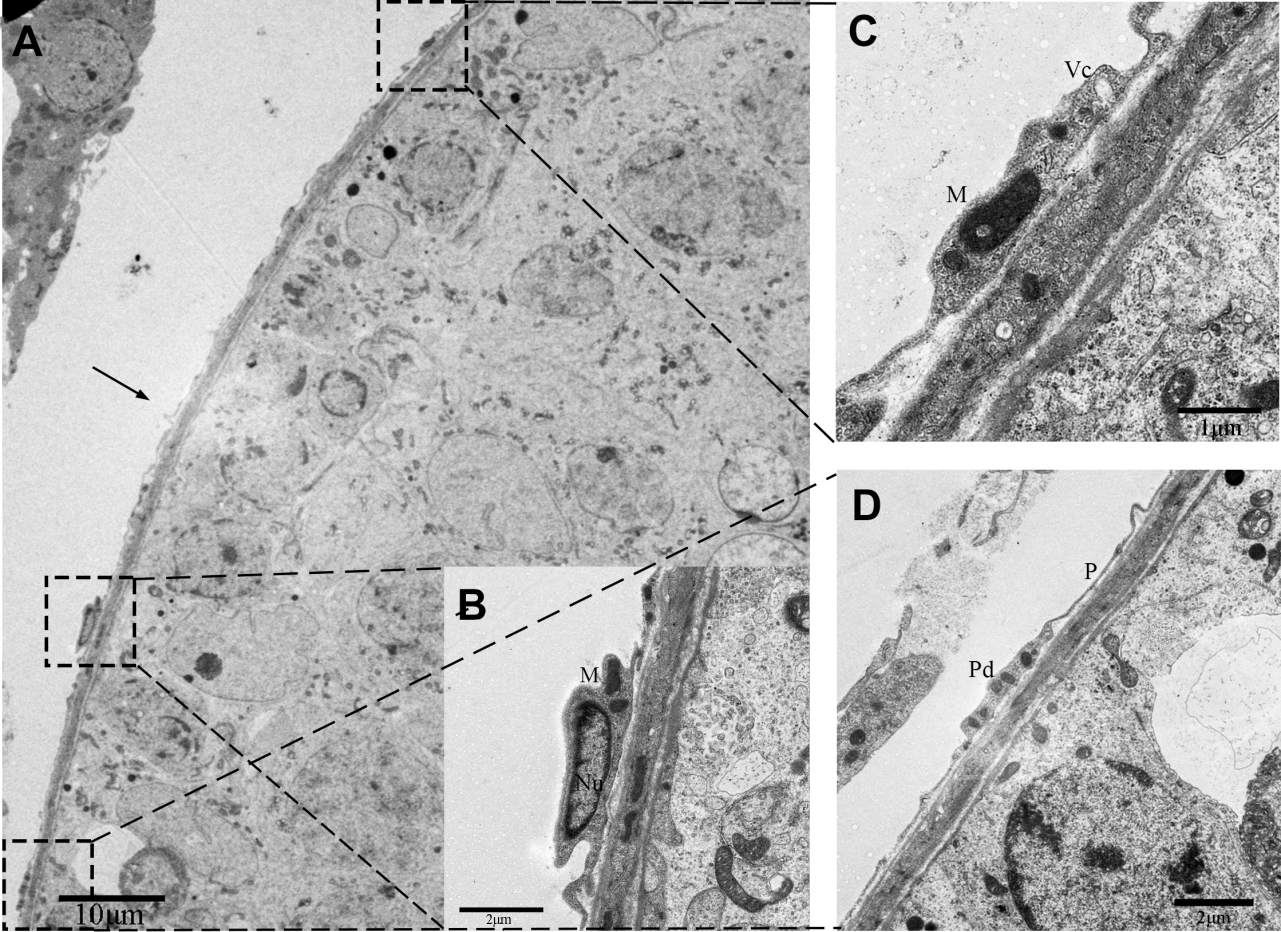
**TEM photograph of a telocyte in rat testis.** (**A**) The telocyte is located surround the seminiferous tubule (indicated by the arrow) and shows a distinct cytoplasmic process with a (**C**) podom containing mitochondria and the vesicles and (**D**) podomer.(**B**) The cell body contains small amount of cytoplasm with mitochondria around the nucleus. Higher magnification illustrates the rectangular area. Nu: nucleus; M: Mitochondria;Vc: vesicles; Pd: Podom; P: podomer. Scale Bar = **A**: 10μm; **B**: 2μm; **C**: 1μm; **D**: 2μm.

TEM images showed that PMC surround the seminiferous tubules (ST; Figure 1A). The PMC cells contained concertina-shaped nuclei, abundant actin filaments, mitochondria, Golgi apparatus and vesicles (Figure 1B–1C). We also observed a thin layer of cells on the outside of the PMC layer that showed long, thin, moniliform cytoplasmic prolongations (Figure 1A, 2A).

These cells with long moniliform projections exhibited features that matched the criteria for classic TCs proposed by Popescus and Faussone-Pellegrini [2]. The cell bodies of these cells were relatively small with sparse cytoplasm containing several mitochondria surrounding the nucleus. The nuclei of these cells were moderately heterochromatic at the periphery (Figure 2B). Additionally, the cellular processes of the TCs or telopodes extended away from the cell body and contained both thin segments (podomers) and dilated bead-like regions (podoms; Figure 2D). The podoms contained both mitochondria and vesicles (Figure 2C).

**Figure 3:**
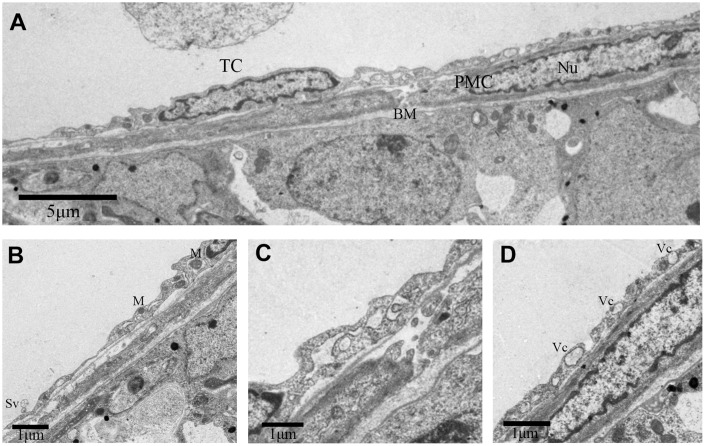
**TEM photograph of a telocyte in rat testis.** (**A**) Telocyte overlays the peritubular myoid cell in the lamina propria of seminiferous tubule. (**B**, **D**) Cytoplasmic process shows long extensions with (**B**) mitochondria and (**D**) vesicles. (**C**) The telopode seems more developed, with winding, thickened and dichotomous feature. TC: Telocyte; PMC: Peritubular myoid cell; Bm: Basement membrane; Nu: Nucleus; M: Mitochondria; Sv: Secretory vesicle; Vc: Vesicles. Scale Bar = **A**: 5μm, **B**–**D**:1μm.

**Figure 4:**
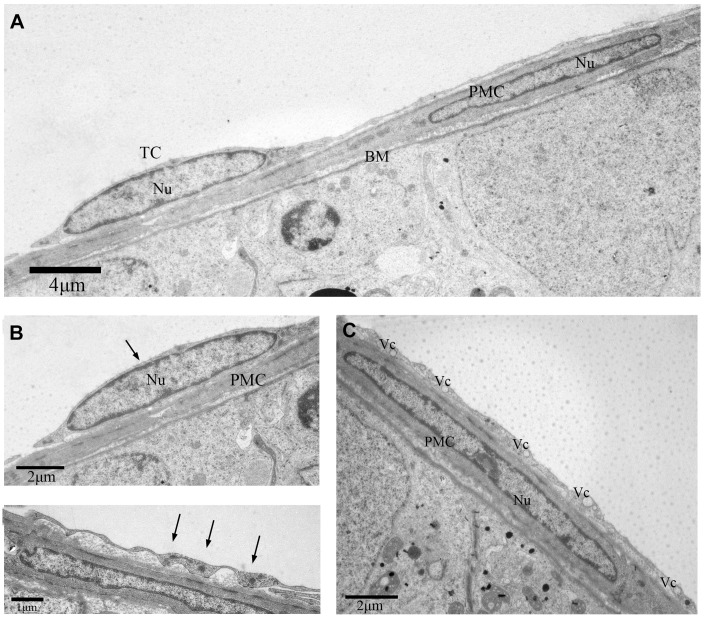
**TEM photograph of another telocyte in the rat testis.** (**A**) The telocyte surrounds the seminiferous tubule and the peritubular myoid cell.(**B**) The telocyte shows distinctly elongated nucleus (indicated by the arrow) with a small amount cytoplasm. (**C**) The cytoplasmic process shows few attachment plaques (indicated by the arrows). (**D**) The cytoplasmic prolongation shows many vesicles. TC: Telocyte; PMC: Peritubular myoid cell; Nu: Nucleus; Bm: Basement membrane; Vc: Vesicles. Scale Bar = **A**: 4μm; **B**:2μm; **C**: 1μm; **D**: 2μm.

We also discovered some other TCs in the outer-layer. These TCs showed a large, elongated nucleus with irregular boundary (Figure 3A). The telopodes in this variant of TCs was more developed with winding, thickened and dichotomous feature (Figure 3C), and contained several mitochondria and vesicles (Figure 3B, 3D). In contrast, some TCs contained a smooth elongated nucleus with scarce perinuclear cytoplasm (Figure 4A, 4B). In these TCs, telopodes contained many vesicles (Figure 4D), and the cells showed attachment plaques (Figure 4C).

**Figure 5:**
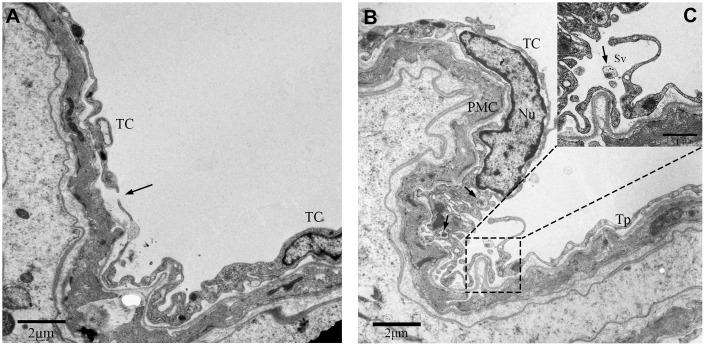
**TEM photograph showing two neighboring telocytes in the rat testes.** (**A**) Telopodes (Tps; indicated by arrow) and (**B**) small secretory vesicles (indicated by the arrows) of the two telocytes are shown.(**C**) Higher magnified image shows the area between the two telocytes. Note: TC: Telocyte; PMC: Peritubular myoid cells; Nu: Nucleus; Tps: Telopode; Sv: Secretory vesicle. Scale Bar = **A**–**B**: 2μm.

**Figure 6:**
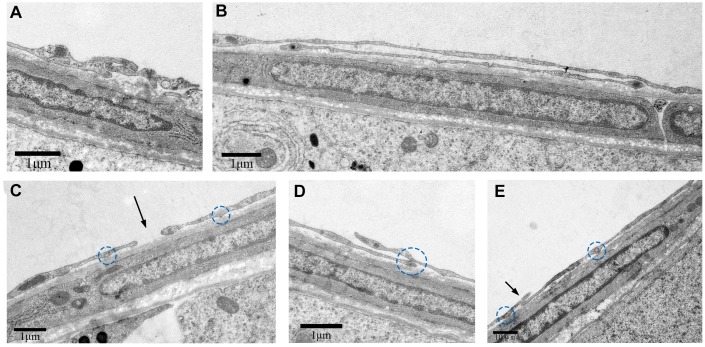
**TEM photograph shows different types of junctions formed by Tps with other cells in the rat testis.** Physical contacts made by Tps with adjacent cells through (**A**) Mortise and tenon joint, (**B**) long overlapped structure, (**C**) closeness (indicated by the arrow), (**D**, **E**) short overlapped structure and punctate contact (indicated by the circles). Scale Bar = **A**–**D**: 1μm; **E**: 10μm**.**

We further observed that these TCs were adjacent to each other. The telopodes (Tps) from neighboring cells were in physical contact with each other (Figure 5A). Several secretory vesicles were observed between the two Tps, suggesting home-cellular communication or exchange of intracellular material between the adjacent TCs through secretory vesicles (Figure 5B, 5C). As shown in Figure 6, various types of junctions were formed by Tps among the contiguous TCs, including the mortise and tenon joint (Figure 6A), punctiform structure (Figure 6B), short overlapped structure with punctate contact (Figure 6D, 6E), and closeness (Figure 6C). Based on these findings, we speculate that TCs surround the ST and communicate with each other through diverse intercellular connections.

### IHC analysis of telocytes in rat testicular interstitium

**Figure 7:**
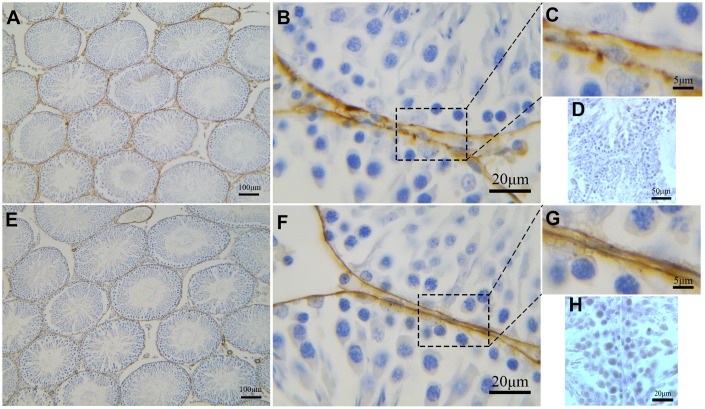
**Immunohistochemical analyses of peritubular tissue in the rat testis with anti-αSMA and anti-CD34 antibodies.** (**A**–**C**) CD34 positive staining (brown) surrounds the seminiferous tubules. Higher magnification (**C**) shows segmental pattern of CD34 staining. (**E**–**G**) αSMA positive staining (brown) also surrounds the seminiferous tubules. Higher magnification (**G**) shows smooth uniform staining pattern. Higher magnification illustrates the rectangular area. (**D**, **H**) shows negative contral group of rat testis. Scale Bar = **A**, **E**: 100μm; **B**, **F**: 20μm; **C**, **G**: 5μm; **D**: 50μm; **H**: 20μm.

Low magnification images of IHC stained specimens show both CD34-positive and α-SMA-positive cells surrounding the entire ST (Figure 7A, 7E). However, at high magnification, positive CD34 staining shows distinct segmentation with the dilated parts showing strong CD34 positivity (Figure 7B, 7C). On the other hand, uniform positive α-SMA staining is observed throughout the smooth layer surrounding the ST (Figure 7F, 7G). Negative control group of rat tesits were also shown (Figure 7D, 7H).

### Double IF analysis of telocytes in rat testicular interstitium

**Figure 8:**
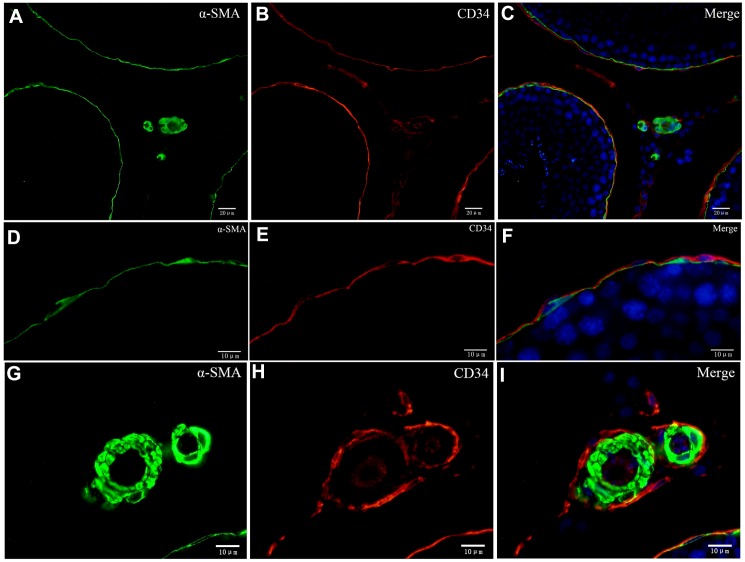
**Double immunofluorescence of telocytes in the rat testis with anti-αSMA and anti-CD34 antibodies.** (**A**–**F**) Immunofluorescence staining shows positive αSMA (**A**; green) and CD34 (**B**; red) staining of the inner and the outer layers surrounding the seminiferous tubules. (**G**–**I**) Immunofluorescence staining shows positive CD34 staining (red) around the blood vessel and in endothelial cells, and positive αSMA staining (green) on the vessel wall. Scale Bar = **A**–**C**: 20μm; **D**–**I**: 10μm.

To explore whether both CD34 and α-SMA mark the same layer of cells, we performed double-immunofluorescence. Our data suggest that distinct layers surround the ST. The inside layer is positive for α-SMA and the outside layer is positive for CD34 (Figure 8A-F). The walls of the blood vessels stain positive for α-SMA while the cells surrounding the blood vessels and the endothelial cells stain positive for CD34 (Figure 8G-I). In conjunction with the results from the TEM analyses, our data suggests that cells with diverse ultrastructure were all positive with CD34. This confirmed that all of the cells were TCs.

**Figure 9:**
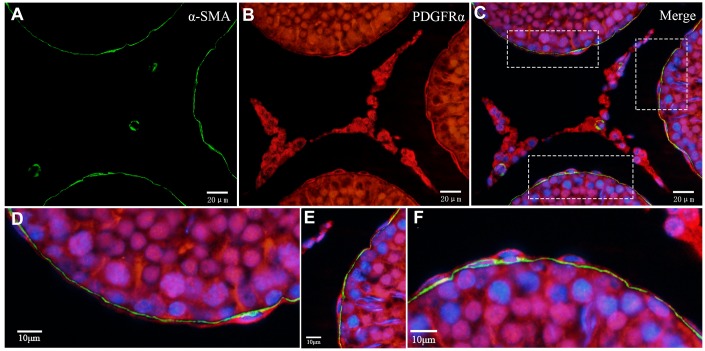
**Double immunofluorescence staining of telocytes in the rat testis with anti-αSMA and anti-PDGFRα antibodies.** (**A**–**C**) Immunofluorescence images show positive αSMA (green) and PDGFRα (red) staining inside/outside as continuous layers around the seminiferous tubule. (**D**–**F**) Immunofluorescence images show higher magnification illustrates the rectangular area in (**C**). Scale Bar = **A**–**C**: 20μm; **D**–**F**:10μm.

**Figure 10:**
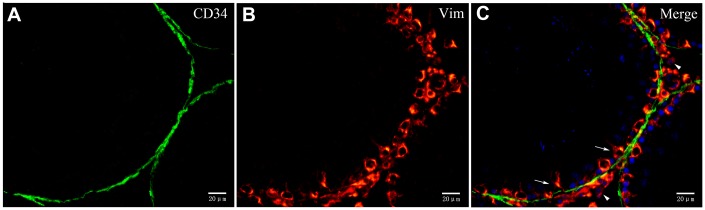
**Double immunofluorescence staining of telocytes in rat testis with anti-CD34 and anti-vimentin antibodies.** (**A**–**C**) Immunofluorscence staining shows that CD34 (green) and Vimentin (red) do not co-localize around the seminiferous tubule. Vimentin is expressed on Sertoli cells (pointed by arrows) and Leydig cells (pointed by triangles). Scale Bar = **A**–**C**: 20μm.

We also observed that the layer enclosing the ST was PDGFRα positive (Figure 9B). Double IF staining showed that the inside layer is PMC stained positive for α-SMA, and the outer layer is TCs stained positive for PDGFRα (Figure 9A-C). This confirmed the existence of TCs in the outer layer surrounding the ST. Furthermore, Leydig cells and Sertoli cells also expressed PDGFRα (Figure 9B). Double-staining for vimentin and CD34 showed that vimentin staining was negative in the layer of cells surrounding ST that were CD34 positive, but positive vimentin staining was observed in the Sertoli and Leydig cells (Figure 10A-C). This suggested that the rat testicular TCs were vimentin negative.

**Figure 11:**
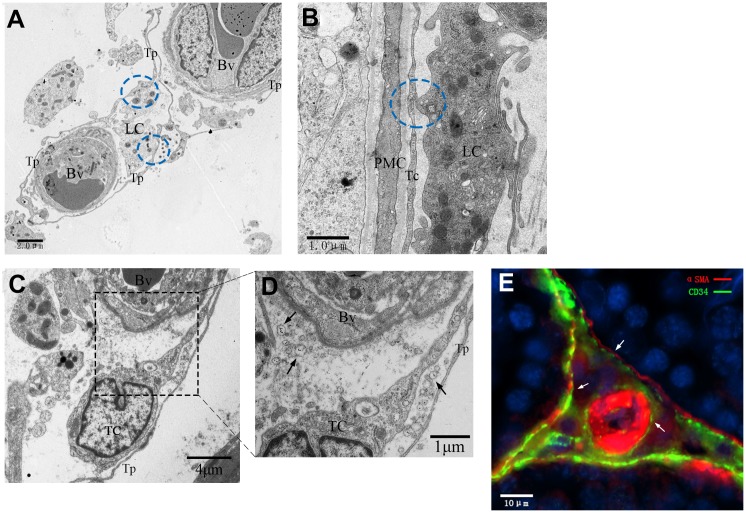
**Interactive network of cells including telocytes in the inter-tubular space of rat testes.** (**A**–**D**) Transmission electron micrographs show that telocytes form interactive networks by extending their telopodes to connect with Leydig cells and surrounding blood vessels (**A**, **C**) through various secretory vesicles (**D**, indicated by the arrows). The circles highlight the direct contact between telopodes and Leydig cells (**A**–**B**). (**E**) Immunofluorescence staining of CD34 (green) and αSMA (red) shows telocytes surrounding the PMC and the blood vessel. Arrows show the telocytes. Higher magnification illustrates the rectangular area. Tp: telopode; Bv: Blood vessel; LC: Leydig cells; PMC: peritubular myoid cells; TC: telocyte. Scale Bar = **A**: 2μm; **B**: 1μm; **C**: 5μm;**D**: 1μm; **E**: 10μm.

Furthermore, TCs were also observed in the intertubular stroma, which suggested that they formed an interactive network with other cells by spreading their processes to surround the entire capillary and the Leydig cells (Figure 11A, 11C). Numerous extracellular vesicles were detected between the telopodes and the vessel wall (Figure 11D). Moreover, TCs located in the outer layer of the PMC were in close membrane-to-membrane contact with the nearby Leydig cells via their telopodes (Figure 11B). These ultrastructural features were in accordance with the immunofluorescence data, which showed that CD34-positive cells established a network structure connecting the ST, blood vessels and the Leydig cells (Figure 11E).

## DISCUSSION

There are very few studies that have focused on the location and role of TCs in the male reproductive system. According to our best knowledge, no study so far has reported on telocytes in the rat testicular interstitium. Previously our group and Marini M et al (2018) reported existence of TC in testis of Chinese soft-shelled turtle [19] and adult human [20]. Ultrastructural findings of these studies show that TCs form the outer cell layer around the PMCs and establish cellular connections with other cells like the Leydig cells [19–20]. Our results in the rat testis are consistent with these findings in the turtle and human testis. However, we observe a single layer of PMC in the rat testis compared to multilayered PMC around the ST in the human testis. It is clearer to distinguish TCs from peritubular tissues due to a single layer of PMC in rat is excluded.

In the present study, all the TCs share some common features. For example, they all possess long and moniliform prolongations containing mitochondria and vesicles. Furthermore, some TCs resembled a myofibroblast morphologically though they had a comparatively smaller cell body and longer telopodes with some attachment plaques. In the human gallbladder, a hybrid type of PDGFRα-positive/α-SMA-positive TCs are present that differ from typical TCs because they have a larger cell body and attachment plaques that resemble myofibroblasts [21].

TCs have the potential to carry signals over long distances through their prolongations [22]. A previous study showed that telopodes have direct membrane-to-membrane contacts through nano-contact, Puncta adherentia minima and gap junctions [4]. The term "stromal synapse" has been used to describe these synaptic connections between TCs and neighboring cells, which can be concluded as juxtacrine (a chemical synapse) and corresponding to an exosome-based mechanism [2]. Moreover, TCs form intercellular connections with the underlying PMCs. The PMCs provide structural support to ST, play an important role in establishing the blood-testis barrier, and their contractility is involved in the transport of spermatozoa and the testicular fluid [23–24]. In the present study, we observed cellular connections among the telocytes, and between the telocytes and other cells. Therefore, we hypothesize that telocytes and their telopodes play an important role in the intracellular communication within the testicular interstitium [25–26].

We performed IHC and double-IF to investigate the functional activity of TCs and distinguish them from other cells. While TCs do not have specific immunophenotypic characteristics, several cell surface markers have been reported on these cells based on their ability to bind different antibodies against known cell surface molecules [27–28]. The TCs are regarded as an immunophenotypic mosaic because of their diverse phenotype, and this depends on their location and state of activation or quiescence [21, 29–30]. Currently, co-expression CD34 and vimentin or CD34 and PDGFRα are used toidentify TCs [31]. In current study results, CD34 and PDGFRα co-expressed in rat testis TCs, which is in coincidence with the findings of TC in human testis. [20].

The PMC’s are strongly positive for the α-SMA cell surface marker, which confirms their smooth muscle myoid cell-like characteristics [32]. Double-IF analysis showed that ST was surrounded by two layers of cells: the inner layer of cells were thePMCs, which were labeled by antibodies against α-SMA, and the outer layer of cells were the TCs, which were stained by fluorescent antibodies against CD34 and PDGFRα. This suggested that TC’s probably co-express CD34 and PDGFRα. Both double IF and TEM data put together suggests that the rat-derived TCs are double positive for CD34 and PDGFRα cell surface markers. We also observed that the rat testis TCs were CD34-positive/vimentin-negative. Vimentin is expressed in mesenchymal cells including fibroblasts, endothelial cells, and myofibroblasts [33]. In previous studies, TCs have been shown to express vimentin in most organs [6, 34], thereby suggesting their homology with mesenchymal cells. Our study shows that TCs do not express vimentin in rat testis, which suggests that they may be of a different origin than previously characterized TCs. Therefore, there is great scope for future studies regarding the origin and the functional mechanisms of TCs in the rat testis.

Both TEM and double-IF data showed that CD34-positive cells formed an interstitial network structure. Peritubular tissue is regarded as the channel for exchanging chemical substances between the ST and the interstitium; androgens also diffuse to the ST through the peritubular tissue [12, 25, 35]. Current research on the local regulatory mechanisms of spermatogenesis mainly focuses on the interactions between SC, PMC, Leydig cells and spermatogenic cells [15, 17, 36]. Since there are no physical connections between Leydig cells and the PMCs in adult male testes [16], it is possible that interactions occur in a paracrine manner or are mediated by other cells. Our data suggests that TCs may be may be involved in the communication between the Leydig cells and the PMCs.

In conclusion, we confirm the existence and distribution of TCs in the rat testis and describe their distinct connection forms. Rat testis TCs form an interactive network connecting the PMCs, Leydig cells and the blood vessels. This study provides a novel structural and functional perspective for the cellular components of the testicular interstitium and improves our understanding of the physiological aspects of testis. The potential function of the TCs and the interactions among different cellular components in the testis requires further investigation.

## MATERIALS AND METHODS

Sprague-Dawley (SD) male rats (n = 10 and 3 months old) weighing 180-200 g were purchased from Qinglong Mountain Animal Breeding Farm, Nanjing, Jiangsu Province, China. They were provided with food and water ad-libitum, and allowed to acclimatize for 10 days under pathogen-free conditions. After 10 days, the rats were anaesthetized by intraperitoneal injection of pentobarbital sodium (60 mg/kg) and euthanized by cervical dislocation. Testes samples were collected immediately and processed further. All animal-related protocols were approved by the Science and Technology Agency of Jiangsu Province (SYXK (SU) 2017-0007).

### Transmission Electron Microscopy (TEM)

The testes specimens were cut into small parts and fixed overnight at 4°C in 2.5% glutaraldehyde in0.1M Phosphate buffered saline (PBS, pH 7.4). Then, the fixed tissue samples were rinsed in PBS and incubated for one hour at room temperature in buffered 1% osmium tetroxide (Polysciences Inc. Warrington, PA, USA). Then, after washing in 0.1M PBS (pH 7.4), the samples were dehydrated using ascending concentrations of ethyl alcohol (50%, 70%, 80% and 100%), permeabilized with a propylene oxide–Araldite mixture, and then embedded in Araldite. Ultrathin sections (50 nm) were cut using an ultramicrotome (ReichertJung, Wien, Austria) and mounted on copper coated grids. The specimens were stained with 1% uranyl acetate and Reynold’s lead citrate for 20 mins. The processed specimens were examined under a transmission electron microscope (TEM; Hitachi H-7650, Japan) and photographed using a high-resolution 16 mega-pixel digital camera connected to the TEM.

### Immunohistochemistry (IHC)

The rat testes tissue samples were fixed in 10% formalin, embedded in paraffin, and cut into 6 μm thin sections. The tissue sections were deparaffinized and then rehydrated with decreasing concentrations of ethyl alcohol (100%, 100%, 95%, 95%, 85% and 75%). Then, endogenous peroxidaseactivity was blocked by incubating the samples in 3% hydrogen peroxide followed byantigen retrieval. The specimens were then blocked with 5% bovine serum albumin (BSA) in room temperature for 30 min, followed by incubation with rabbit anti-CD34 (1:100 dilution; catalog no. BA3414; Boster, Wuhan, China), mouse anti-αSMA (1:100 dilution; catalog no. BM0002; Boster, Wuhan, China) antibodies at 4°C for 24 hours. Negative contral group was performed by 0.01 PBS (pH=7.4) at the same condition. After washing, the sections were incubated with biotinylated anti-rabbit/anti-mouse IgG (Boster Bio-Technology, Wuhan, China) for one hour at room temperature. The sections were then incubated with avidin-biotinylated peroxidase complex. Peroxidase activity was determined by staining with diaminobenzidine (DAB, Boster Bio-Technology, Wuhan, China). The nuclei were stained with hematoxylin.

### Double Immunofluorescence (IF)

The rat testes tissue paraffin sections were deparaffinized followed by antigen unmasking in sodium citrate buffer. Then, the tissue sections were incubated with 1% bovine serum albumin (room temperature for 30 min) to block non-specific antibody binding. Following this, the samples were incubated at 4°C overnight with the following primary antibodies: rabbit anti-CD34 (1:100 dilution; catalog no. BA3414; Boster, Wuhan, China); rabbit anti-PDGFRα (1:100 dilution; catalog no. BA0275; Boster, Wuhan, China); mouse anti-vimentin (1:100 dilution; catalog no. BA0135; Boster, Wuhan, China); and mouse anti-αSMA (1:100 dilution; catalog no. BM0002; Boster, Wuhan, China). Then, after washing with 0.1M PBS (pH 7.4), samples were incubated with Alexa Fluor-488-conjugated goat anti-rabbit IgG (1:150 dilution; catalog no. RBaf48801; Fcmacs, Nanjing, China) and Tritc-conjugated goat anti-mouse IgG (1:150 dilution; catalog no. BA1089; Boster, Wuhan, China), Tritc-conjugated goat anti-Rabbit IgG (1:150 dilution; catalogno. BA1090; Boster, Wuhan, China) and Alexa Fluor-488conjugated goat anti-Mouse IgG (1:150 dilution; catalog no. BA1126; Boster, Wuhan, China) secondary antibodies for two hours at 37°C. Nuclei were counterstained with 4′,6-diamidino-2-phenylindole (DAPI) (catalog no. 13G04A76; Boster, Wuhan, China). After mounting with glycerin, sections were observed under an Olympus BX53 microscope and fluorescent images were captured with an Olympus DP73 digital color camera.
